# Identification of KIFC1 as an independent prognostic marker in renal clear cell carcinoma correlates with tumor proliferation and immune infiltration

**DOI:** 10.1038/s41598-023-43732-4

**Published:** 2023-10-03

**Authors:** Bin Du, Jia Wang, Jinping Zheng, Jing Huo, Pu Wang

**Affiliations:** 1 Center of Healthy Aging, Changzhi Medical College, Changzhi, 047500 China; 2 Department of Biology, Changzhi Medical College, Changzhi, 047500 China

**Keywords:** Cancer, Oncogenes, Tumour biomarkers, Tumour immunology

## Abstract

Renal clear cell carcinoma (ccRCC) is the world's most common form of cancer. Up to a third will develop metastases; the 5-year survival rate of the patients was only 14%. Practical prognostic markers remain to be discovered. Kinesin-like protein (KIFC1), a critical factor in maintaining the stability of the microtubule system, has significant prognostic value in some tumors. We analyzed the prognostic value, associated signaling pathways, and regulatory mechanisms of KIFC1 in ccRCC through bioinformatics and proteomics. Concretely, both mRNA and protein expression levels of KIFC1 were dramatically upregulated. KIFC1 is an independent prognostic factor for ccRCC. The expression of KIFC1 showed a significant positive correlation (Spearman coefficient > 0.7) with tumor proliferation-related pathways (tumor proliferation, G2/M checkpoint, and DNA replication) and tumor inflammation. Further, intratumoral immune cell analysis revealed that high expression of KIFC1 predicted more infiltration of CD8 + T and CD4 + T cells (*p* < 0.001). However, there was a significant positive relationship between CD8 + T cells and numerous immune checkpoint genes. CD8 + T cells in tumors from the KIFC1 high expression group were at the dysregulated state. High expression of KIFC1 may predict a poor immunotherapy outcome. By proteomics, we analyzed proteins interacting with KIFC1; spliceosome proteins had the most significant enrichment, indicating the new directions for KIFC1 investigation. In conclusion, our study identified KIFC1 as an independent prognostic factor in renal clear cell carcinoma, and the associated processes involved tumor proliferation and immune infiltration. KIFC1 had a close relationship with spliceosome proteins; it may be a new research direction.

## Introduction

Renal cell carcinoma (RCC) is the most common cancer in the world, with over 400,000 cases diagnosed annually. Most RCC cases occur between the ages of 60–70, and men are more likely to develpo the disease than women^1^. Approximately 70% of individuals with RCC are diagnosed with clear cell RCC (ccRCC)^1^. Despite the ability to treat ccRCC early and effectively through surgery or ablative strategies, up to a third will develop metastases^2^, However, only 14% of patients with distant metastases survive five years. As a result, the development of new tumor markers that are prognostically valuable is crucial to the diagnosis and treatment of renal clear cell carcinomas.

Kinesin-like protein (KIFC1) is a critical factor in maintaining the stability of the microtubule system^3^. Numerous tumors showed a poor prognosis in association with KIFC1. Targeting KIFC1 could significantly enhance the lethality of chemotherapeutic drugs for tumors^4^. The underlying mechanisms may be involved in centrosome de-cluster^5^, energy metabolism^6^ and endoplasmic reticulum dysfunction^7^. Mechanistically, the expression of KIFC1 was regulated by TCF-4, the critical regulator of the Wnt/β-catenin pathway, and promotes the transcription of HMGA1^8^. Deletion of KIFC1 results in the degradation of lamin B and A/C, defective spindle assembly, formation of micronuclei, as well as loss of chromosomes during mitosis^5^. Coincidentally, KIFC1 was shown to promote the proliferation of tumor cells by regulating AKT, CENPE, ZWINT, and other pathways^9–11^. Existing data show that high expression of KIFC1 could support the proliferation of tumor cells. Elevated KIFC1 could significantly improve the cell's ability to withstand centrosome amplification and multipolar division and resulting genetic instability.

The molecular function and prognostic significance of KIFC1 in renal cancer remain unclear. In this study, we systematically analyzed KIFC1 expression in tumors and its correlation with tumor prognosis by bioinformatics. Additionally, we analyzed the correlation between the mRNA expression of KIFC1, numerous signal transduction pathways, and immune infiltration. To provide suggestions for the diagnosis and treatment of renal clear cell carcinoma.

## Materials and methods

### Gene expression analysis

In the TCGA dataset (https://portal.gdc.com), RNA-sequencing expression profiles (level 3) for ccRCC were downloaded. The current-release (V8) GTEx datasets were obtained from the GTEx data portal website (https://www.gtexportal.org/home/datasets). The statistical analysis was conducted using R software version 4.0.3 (R Foundation for Statistical Computing, Vienna, Austria). We considered *p*-values less than 0.05 to be statistically significant.

### Gene prognostic analysis.

To compare survival rates between these groups, a log-rank test was used. Time-ROC analysis (version 0.4) was used to determine whether KIFC1 mRNA can predict survival. We used R (foundation for statistical computing 2020) version 4.0.3 to implement all the analysis methods, and *p* values under 0.05 were considered statistically significant.

### Analysis of KIFC1 protein expression

Data obtained from Human Protein Altas (https://www.proteinatlas.org).

### Multivariate prognosis analysis.

To construct the nomogram, we used both univariate and multivariate cox regression analysis. The forest was used to show the *p* value, HR and 95% CI of each variable through ‘forestplot’ R package. We developed a nomogram to predict the X-year overall recurrence based on the results of multivariate cox regression analysis (Supplementary Material).

### Clinical characteristics analysis

Statistical analysis and ggplot2 (version 3.3.2) were completed using R program v4.0.3, *p* value < 0.05 was considered statistically significant.

### Differentially expressed genes and functional analysis

ccRCC expression profiles from RNA-sequencing (level 3) and corresponding clinical information were obtained from the TCGA dataset (http ://portal.gdc.com). To study the differentially expressed mRNA, we used the R package limma . “Adjusted *p* < 0.05 and Log2 (Fold Change) > 1 or Log2(Fold Change) <  − 1” were defined as the threshold for the differential expression of mRNAs. In order to further confirm the functional role of potential targets, GO and KEGG functional enrichment analyses were performed. In order to better understand the carcinogenesis of mRNA, ClusterProfiler package (version 3.18.2) in R was used to analyze potential targets' GO function and enrich the pathways associated with KEGG.

### Correlation analysis of genes and pathways

GSVA package of R software was used to analyze, with parameter 'method = ssgsea'. By Spearman correlation, the correlation between genes and pathway scores was analyzed. The analysis methods and R packages were implemented in R version 4.0.3. Statistical significance was determined at a *p* value of 0.05.

### Immunocyte analysis in tumor

To assess the reliable results of immune score evaluation, we used immuneeconv. It’s an R software package that integrates six latest algorithms, including TIMER, xCell, MCP-counter, CIBERSORT, EPIC and quanTIseq. These algorithms had beeen benchmarked, each had a unique advantage. All the above analysis methods and R package were implemented by R foundation for statistical computing (2020) version 4.0.3 and software packages ggplot2 and heatmap.

### Analysis of immune checkpoint

We selected SIGLEC15, TIGIT, CD274, HAVCR2, PDCD1, CTLA4, LAG3 and PDCD1LG2 as immune-checkpoint-relevant transcripts and analyzed their expression levels. In order to implement all the above analysis methods and R package, we used R foundation for statistical computing (2020) version 4.0.3 using the ggplot2 R package as well as the heatmap R package.

### Tumor immune dysfunction and exclusion analysis

We downloaded RNA-sequencing expression levels and clinical information for ccRCC from the TCGA database (https://portal.gdc.com). A potential immune checkpoint blockade (ICB) response was predicted using the TIDE algorithm^12^.

### Immunofluorescence staining

We cultured HK2 cells on coverslips and fixed them in 4% paraformaldehyde after washing three times in 0.1 M phosphate-buffered saline (PBS) for 3 min each. A bovine serum albumin solution was immersed in the coverslips for 60 min at room temperature and then incubated at 4 °C overnight with an indicated antibody. Coverslips were washed 3 times in PBS for 3 min each, then incubated for 60 min with a secondary antibody conjugated to horseradish peroxidase . Following immunostaining, the cells were examined under a light microscope (Leica, Shanghai Trading Co.Ltd. China).

### LC–MS/MS analysis

Analyses were performed on a Q Exactive HF-X mass spectrometer coupled with a ThermoFisher Scientific Easy LLC 1200. The phosphopeptides were loaded onto a self-packed column using buffer A for the phosphoproteomic study. At a flow rate of 300 nL/min, peptides were eluted over 110 min using a linear gradient of buffer B from 2–40%. MS scans were acquired from m/z 350 to m/z 1800 with a resolution of 60,000 at m/z 200 and an injection time of 50 ms. Afterwards, data-dependent top 15 MS/MS scans with normalized energy 28 were applied with 15,000 resolution at 200 m/z using higher-energy collision dissociation (HCD). Isolation window size was set to 1.6 Th, and dynamic exclusion duration was 30 s.

### PPI network construction and hub module selection

To evaluate protein–protein interactions (PPI), we utilized the Search Tool for the Retrieval of Interacting Genes (STRING) database available at http://www.string-db.org/. This database was also instrumental in quantifying the relationships among the differentially expressed genes (DEGs). Furthermore, we employed Cytoscape software to construct the PPI network. The selection of genes with the highest node score and the strongest connectivity was based on statistical significance, with a threshold of *p* < 0.05^13^.

## Result

### High expression of KIFC1 is associated with poor prognosis in ccRCC

**Figure 1:**
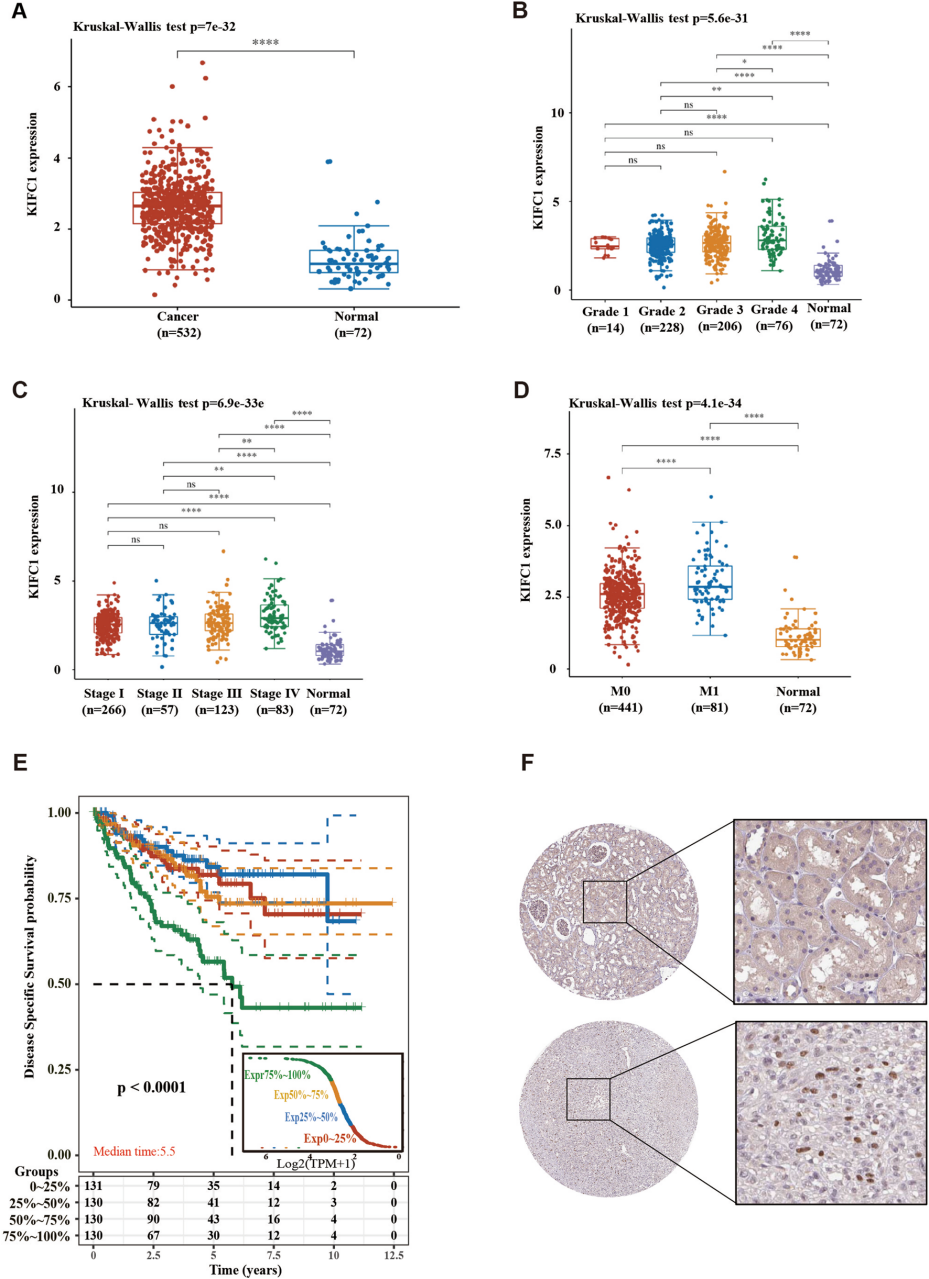
High KIFC1 expression correlates with poor prognosis of KIFC1. (**A**) The expression distribution of KIFC1 in tumor tissues and normal tissues. (**B**) The expression distribution of KIFC1 in tumor grade; (**C**) The expression distribution of KIFC1 in tumor stage; (**D**) The expression distribution of KIFC1 in the primary tumor (M0) and metastases (M1); E: Kaplan–Meier survival analysis of KIFC1 from TCGA dataset, comparison among different groups, quantile was used to group KIFC1 expression; F: Immunohistochemical analysis of KIFC1 in normal and tumor tissues. *p < 0.05, **p < 0.01, ***p < 0.001.

We analyzed the data of 532 tumor tissues and 72 normal tissues in TCGA. The mRNA expression of KIF1 was significantly elevated in RCC tissues compared with that in normal tissues (Fig. 1A). There was no significant difference in the expression of KIFC1 in tumor tissues of different pathological grade and clinical stages (Fig. 1B,C). However, the expression of KIFC1 increased significantly in tumor tissues with metastasis (Fig. 1D). The results of prognosis analysis showed that the high expression of KIFC1 (top 25%) was associated with the poor prognosis of ccRCC (Fig. 1E). A significant increase of KIFC1 expression was observed in tumor tissues relative to healthy tissues (Fig. 1F). With the increased mRNA expression of the KIFC1 gene, the 5-year survival rate and median survival period of ccRCC showed a very significant decrease (*p* < 0.0001). Further, through the public database Human Protein Altas, we preliminarily analyzed the difference in the protein expression of KIFC1 in normal kidney tissue (patient ID: 2530) and ccRCC (patient ID: 848). The immunohistochemistry results showed that the protein expression of KIFC1 was significantly increased in ccRCC. The positive signal of KIFC1 was present in the nucleus, and the proportion of positive cells was not more than 50%.

### Construction of nomogram

**Figure 2:**
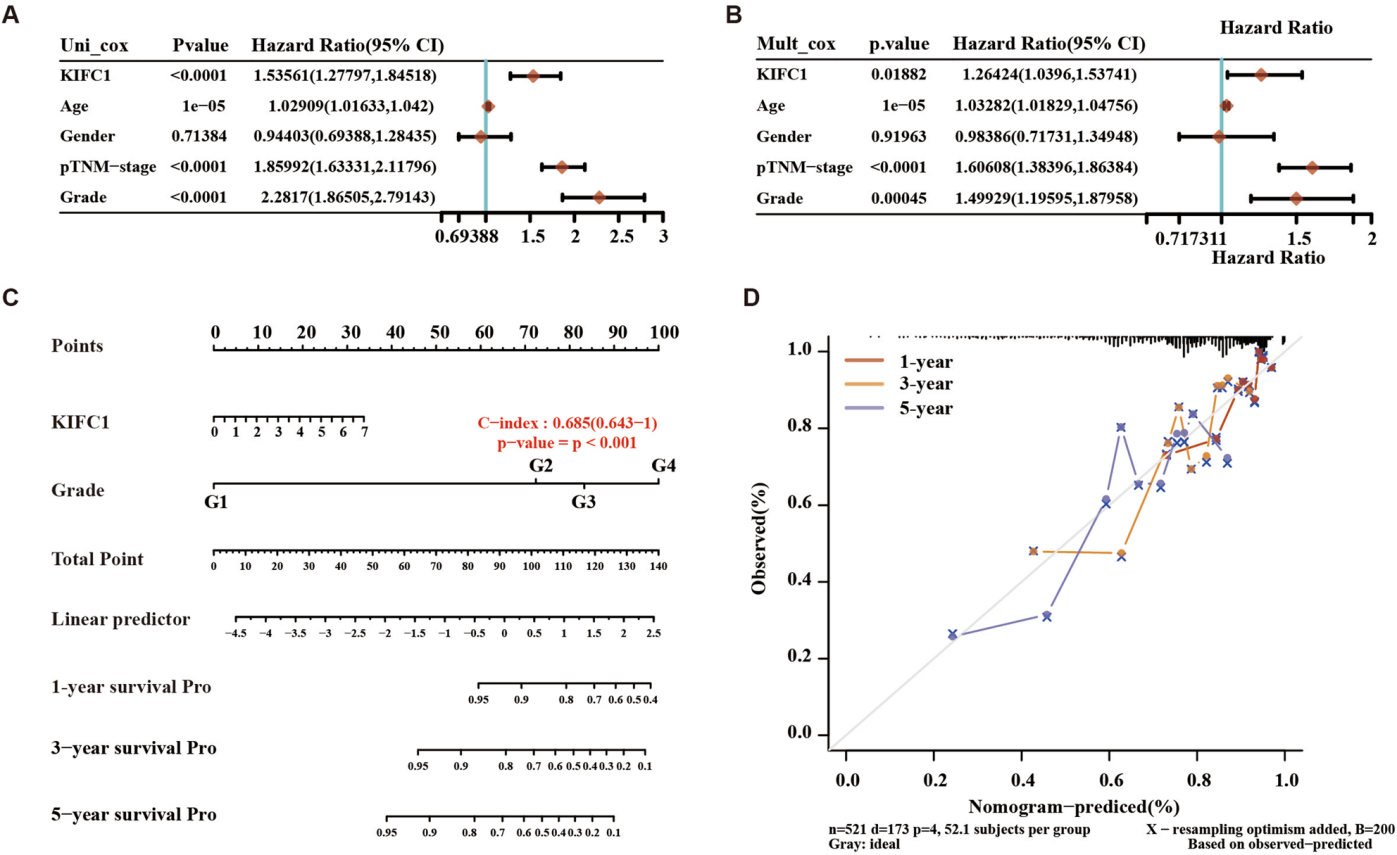
KIFC1 is an independent prognostic factor of ccRCC (**A**) Uni-cox prognosis analysis of KIFC1 in ccRCC; (**B**) multi-cox prognosis analysis of KIFC1 in ccRCC; (**C**) The construction of nomogram. (**D**) The calibration plots for predicting patient 1-year, 3‐year or 5‐year PFS. Nomogram‐predicted PFS is plotted on the x‐axis; observed PFS is plotted on the y‐axis.

To further analyze the correlation between KIFC1 and tumor prognosis. We further construct a nomogram combining the four independent prognostic factors, including the risk score and tumor stage, tumor grade, patients age, and gender, to provide a quantitative method for the clinicians to analyze the *p* value in uni-cox and multi-cox models (Fig. 2A,B). Every patient would get a total point plus each prognostic parameters point, and the higher total points mean a worse outcome for that patient. The results of DCA analysis also demonstrated that our nomogram was of high potential for clinical usefulness (Fig. 2C). Moreover, the calibration curve indicated good performance in the estimation of 1-year, 3-year, and 5-year PFS of the nomogram compared with the estimation of Kaplan–Meier (Fig. 2D).

### Analysis of genes associate with KIFC1

**Figure 3:**
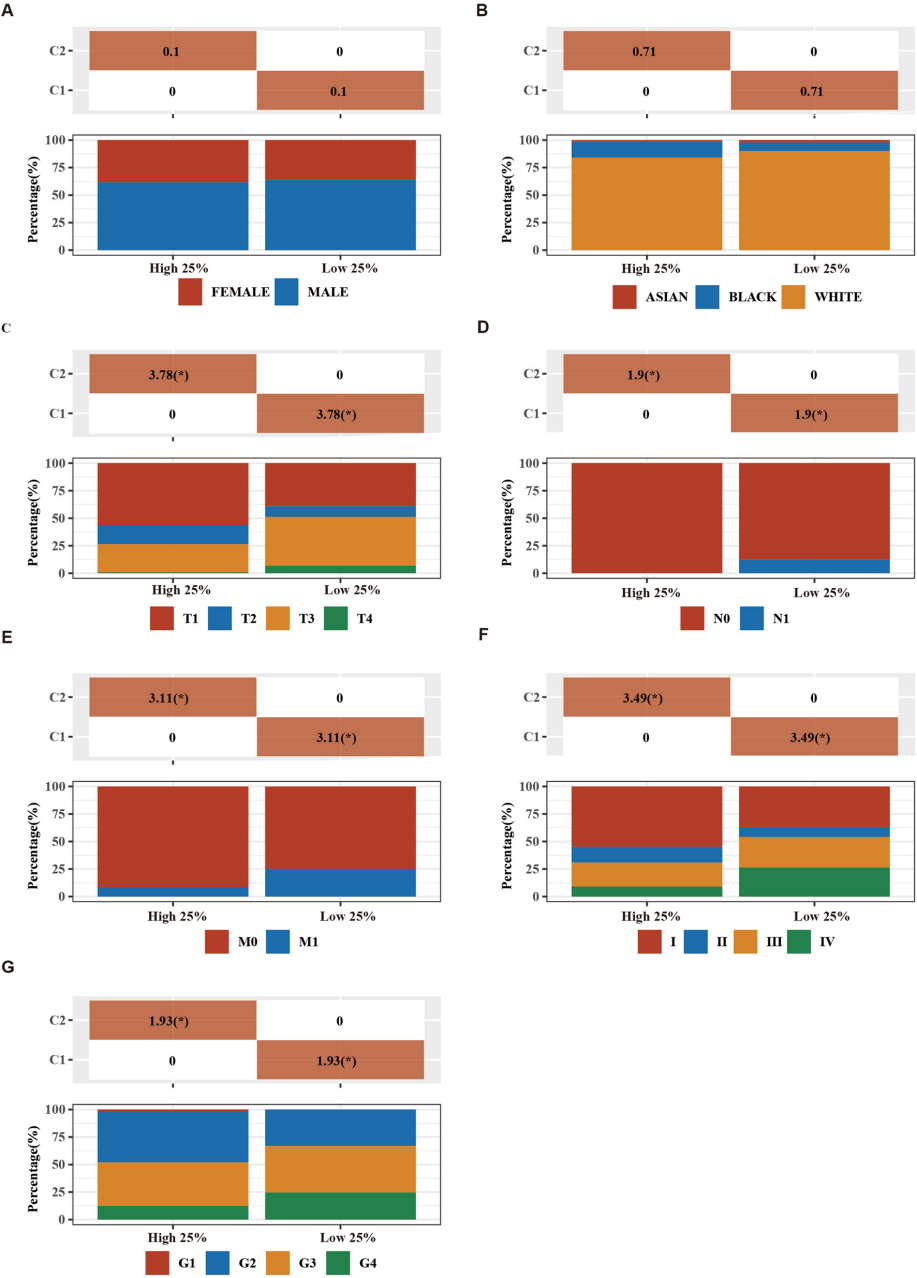
The distribution of clinical characteristics in the samples from different groups. Comparison of gender (**A**), race (**B**), clinical stage of T (**C**), N (**D**), M (**E**), tumor stage (**F**), and grade (**G**) differences between the KIFC1 high expression group and low group. *p < 0.05.

**Figure 4:**
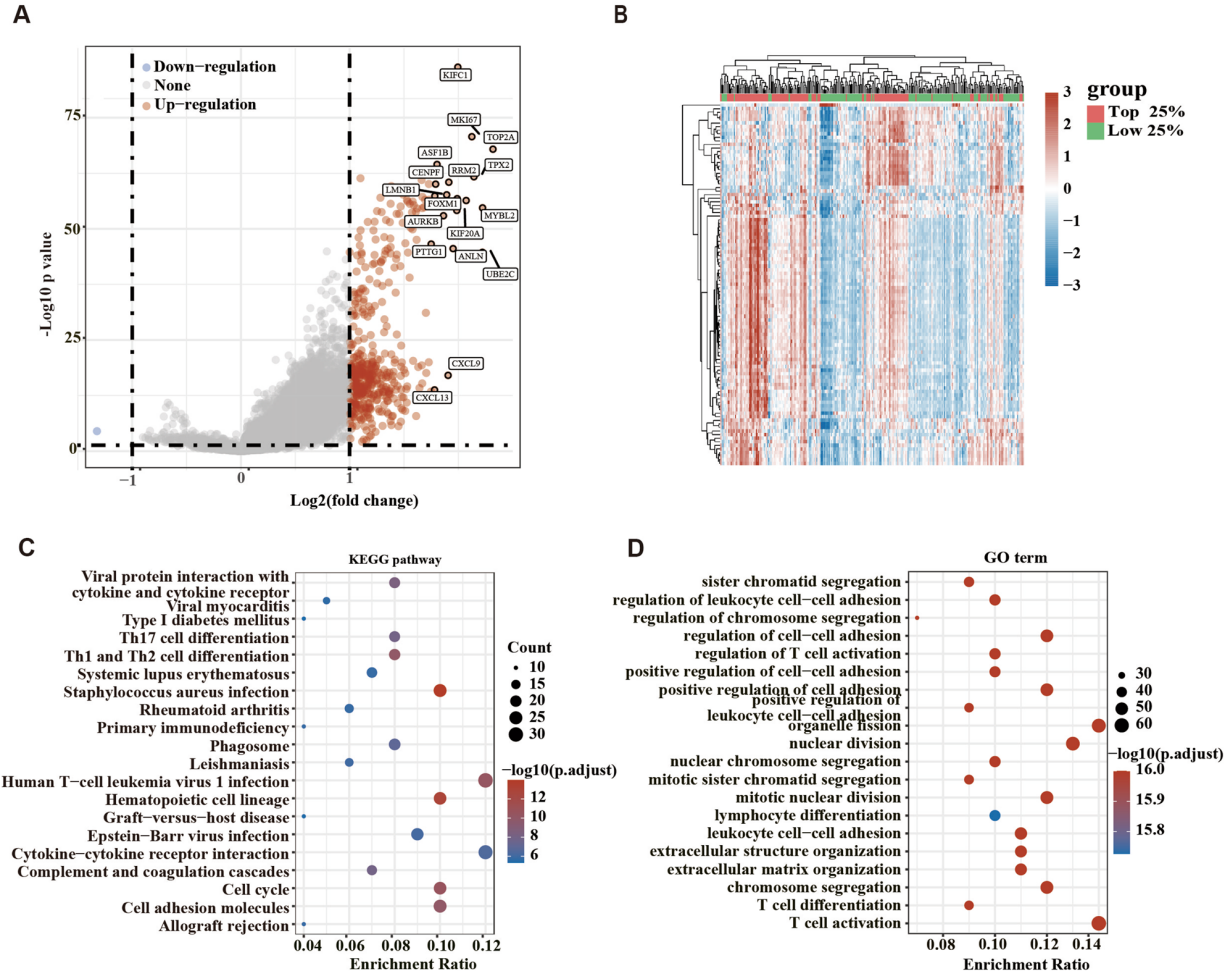
Analysis of KIFC1-related differential genes (**A**): Volcano plot of the genes with a significant difference; (**B**) Heatmap of the genes with a significant difference; (**C**) KEGG analysis of the significantly up regulated genes; (**D**) GO analysis of the significantly up regulated genes.

Further, we took samples of the top 25% and the last 25% of KIFC1 mRNA expression for analysis. There were significant differences in TNM stage, tumor grade, and other indicators between the two groups (Fig. 3). Subsequently, we analyzed the genes significantly different between the KIFC1 high and low expression groups. 441 significantly upregulated genes were identified in the KIFC1 top 25% group compared to the KIFC1 low 25% group (Fig. 4A,B). Through KEGG and GO analysis, we found that the genes significantly associated with KIFC1 mainly focused on cell proliferation and inflammation-related pathways (Fig. 4C,D).

### Analysis of KIFC1 associated pathways

**Figure 5:**
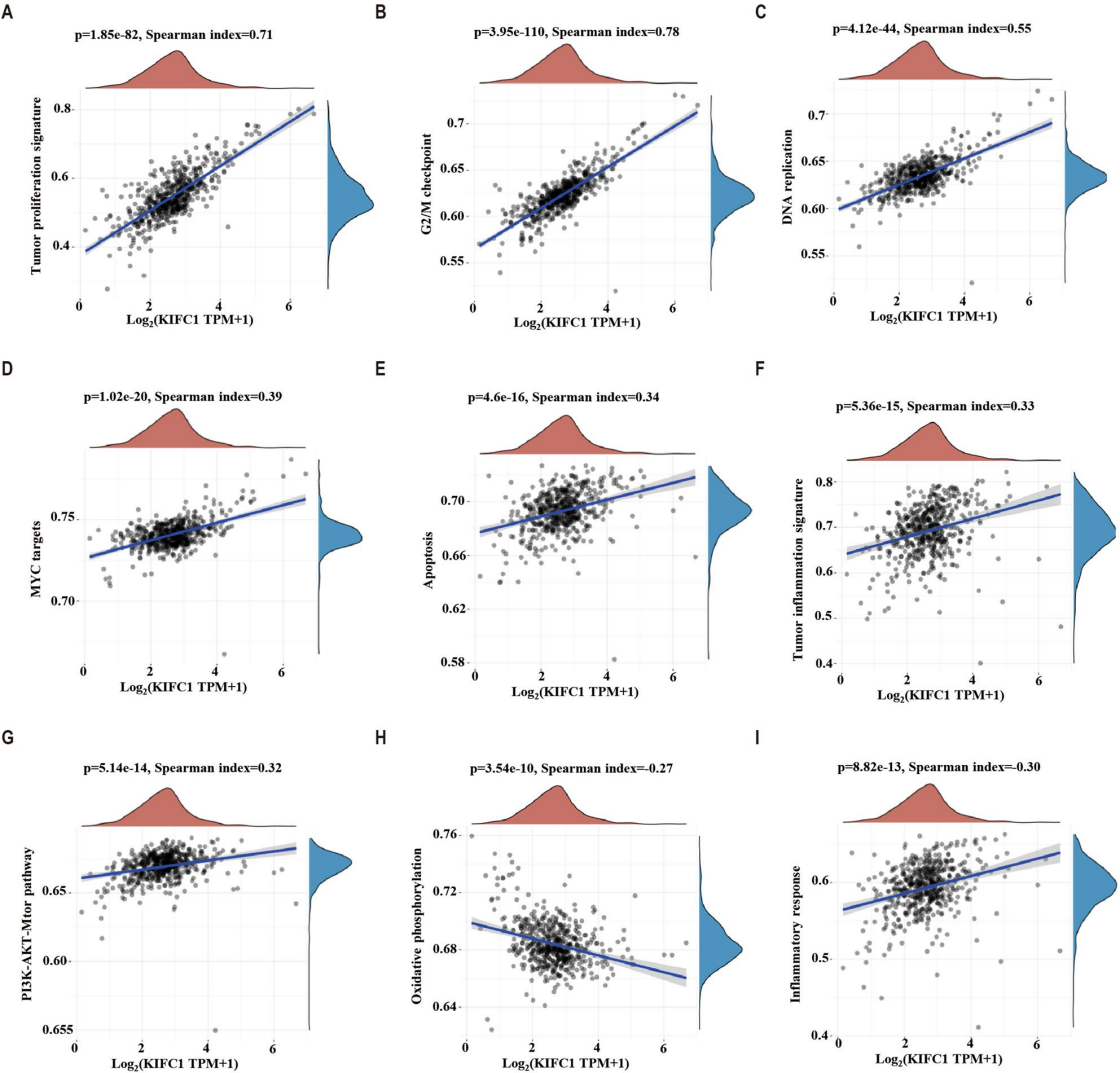
Analysis of KIFC1-related signal transduction pathway. Correlation analysis of tumor proliferation (**A**), G2M checkpoint (**B**), DNA replication (**C**), MYC target genes (**D**), apoptosis (**E**), tumor inflammation (**F**), PI3K-AKT-mTOR (**G**), Oxidative phosphorylation (**H**) and inflammatory response pathways (**I**).

Based on GO and KEGG clustering results, we analyzed the correlation of KIFC1 with tumor-related signaling pathways using the ssGSEA algorithm. Proliferation relative pathways tumor proliferation (spearman = 0.71, *p* < 0.01, Fig. 5A), G2M checkpoint (spearman = 0.78, *p* < 0.01, Fig. 5B), DNA replication (spearman = 0.55, *p* < 0.01, Fig. 5C), MYC targets (spearman = 0.39, *p* < 0.01, Fig. 5D) showed a robust correlation. Inflammation relative pathways tumor inflammation (spearman = 0.33, *p* < 0.01, Fig. 5F) and inflammatory response (spearman = 0.30, *p* < 0.01, Fig. 5I), showed significant correlation. The oxidative phosphorylation pathway showed a significant negative correlation (spearman =  − 0.27, *p* < 0.01, Fig. 5H). Pathways apoptosis (Fig. 5E) and PI3K-AKT-mTOR (Fig. 5G) also showed a significant correlation.

### Intratumor correlation analysis

**Figure 6:**
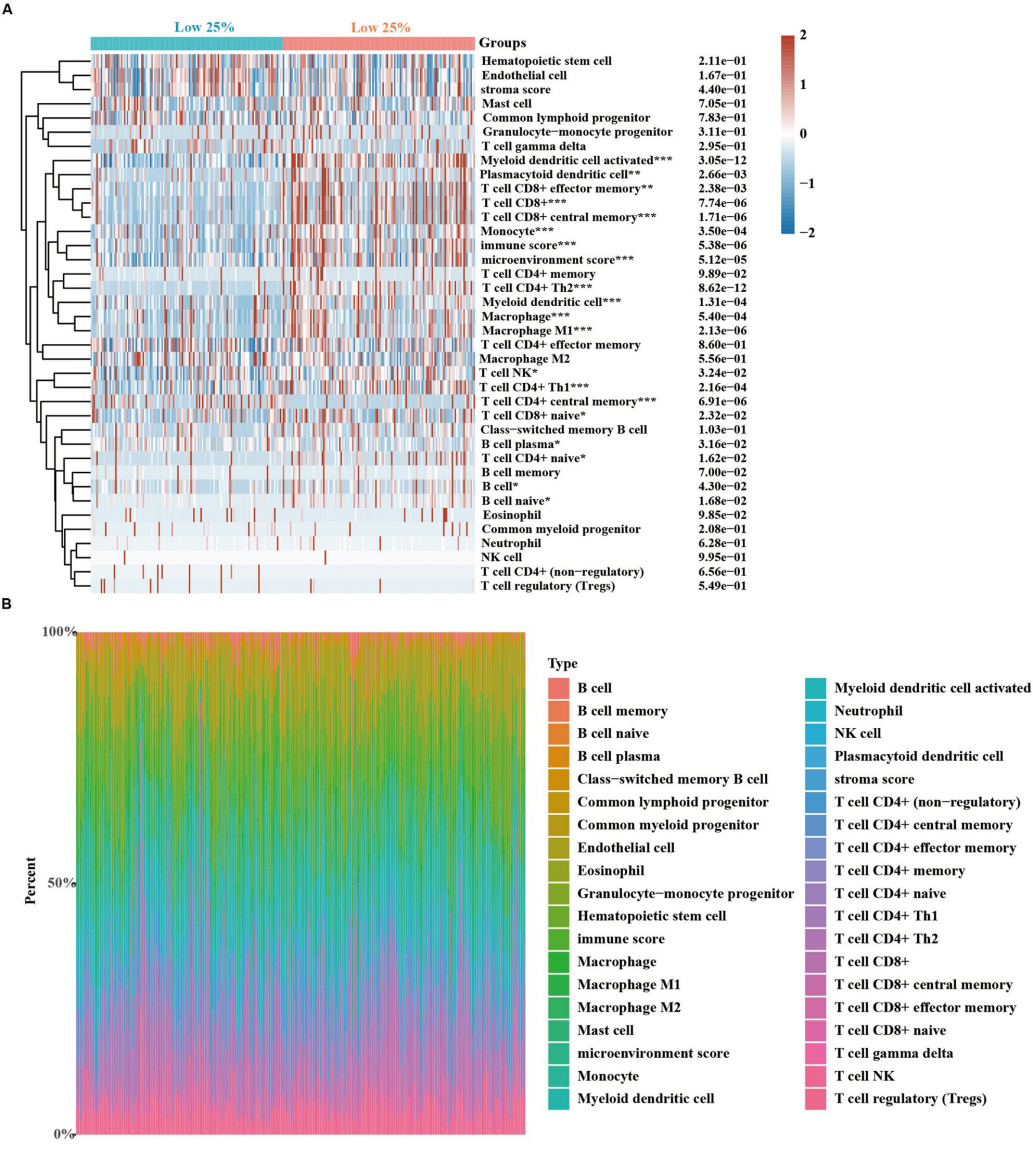
KIFC1 associated immune infiltration in ccRCC. (**A**) Immune cell score heatmap; (**B**) The percentage abundance of tumor-infiltrating immune cells in each sample; the statistical difference of two groups were compared through the Wilcox test *p < 0.05, **p < 0.01, ***p < 0.001.

Because KIFC1 was associated with inflammatory pathways, further, through the xcell algorithm, we analyzed the differences in immune cells between KIFC1 high and low expression groups. We noted the significant differences in the number of T cells and macrophages among a variety of cells (Fig. 6A). Specifically, among T cells, CD8 + T cells and CD4 + T cells were significantly elevated in the group with high KIFC1 expression. For macrophages, only the amount of M1 macrophages was significantly elevated in the KIFC1 high expression group. Overall, T cells represent about 50% of the total immune cells (Fig. 6B).

### Immune checkpoint and ICB response analysis

**Figure 7:**
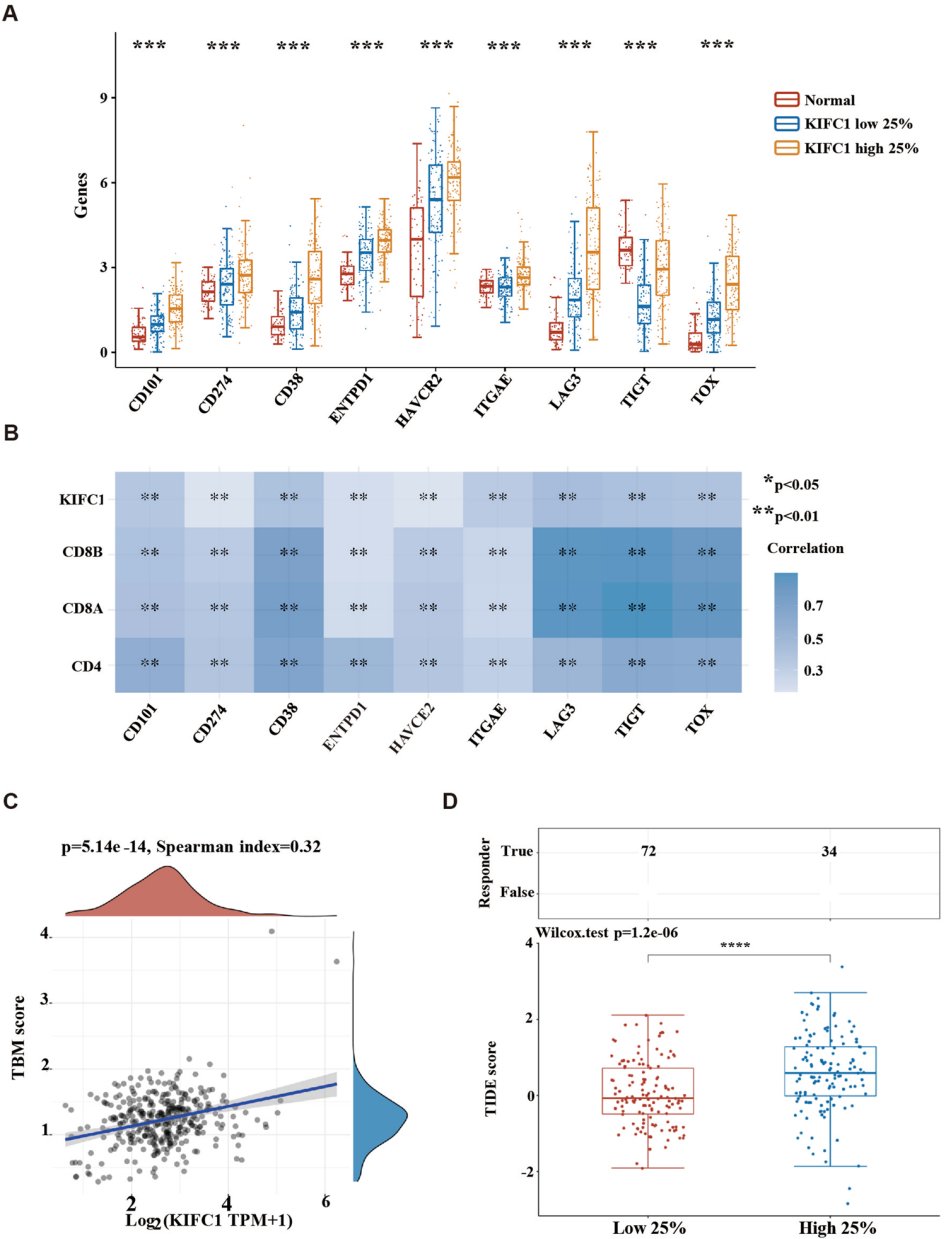
Immune checkpoint and ICB response analysis. (**A**) The distribution of immune response scores in different groups; (**B**) The correlation between CD4, CD8, KIFC1 and immune checkpoint genes; (**C**) Correlation of KIFC1 expression and TMB score; (**D**) TIDE analysis between KIFC1 and low high expression groups. ***p < 0.001, ***p < 0.0001.

Further, we analyzed the expression of common immune checkpoint genes (SIGLEC15, TIGIT, CD274, HAVCR2, PDCD1, CTLA4, LAG3, and PDCD1LG2) in the KIFC1 high versus low expression groups. A highly significant rise was observed for all immune checkpoint genes (Fig. 7A). Because the amount of M2 macrophages in the tumors did not correlate significantly with KIFC1, we suspected an immune dysregulation in ccRCC. The mRNA expression of CD4 and CD8 is significantly correlated with the expression of immune checkpoint genes (Fig. 7B). Significant positive correlation was observed between the mRNA expression of KIFC1 and the tumor mutational burden (TMB) (Fig. 7C). The KIFC1 high expression group had a higher TIDE score, indicating that high expression of KIFC1 predicted a worse response to immune checkpoint blockade therapy (Fig. 7D).

### Identifying proteins interact with KIFC1

Given the above bioinformatics analysis, we found the critical role of KIFC1 in the progression of tumor proliferation and immunity. Investigating the underlying molecular mechanisms is essential to understand their biological functions. First, we investigated the expression and subcellular localization of KIFC1 by immunofluorescence staining. The results showed that the expression of KIFC1 was dependent on cell cycle progression. During interphase, the amount of KIFC1 in the cells was meager. When cells enter prophase, KIFC1 starts localized to the nucleus, and its expression begins to rise. During metaphase and anaphase, KIFC1 fully localizes to the spindle. During progressive telophase, it localizes to chromosomes and telophase microtubule bundles.

**Figure 8:**
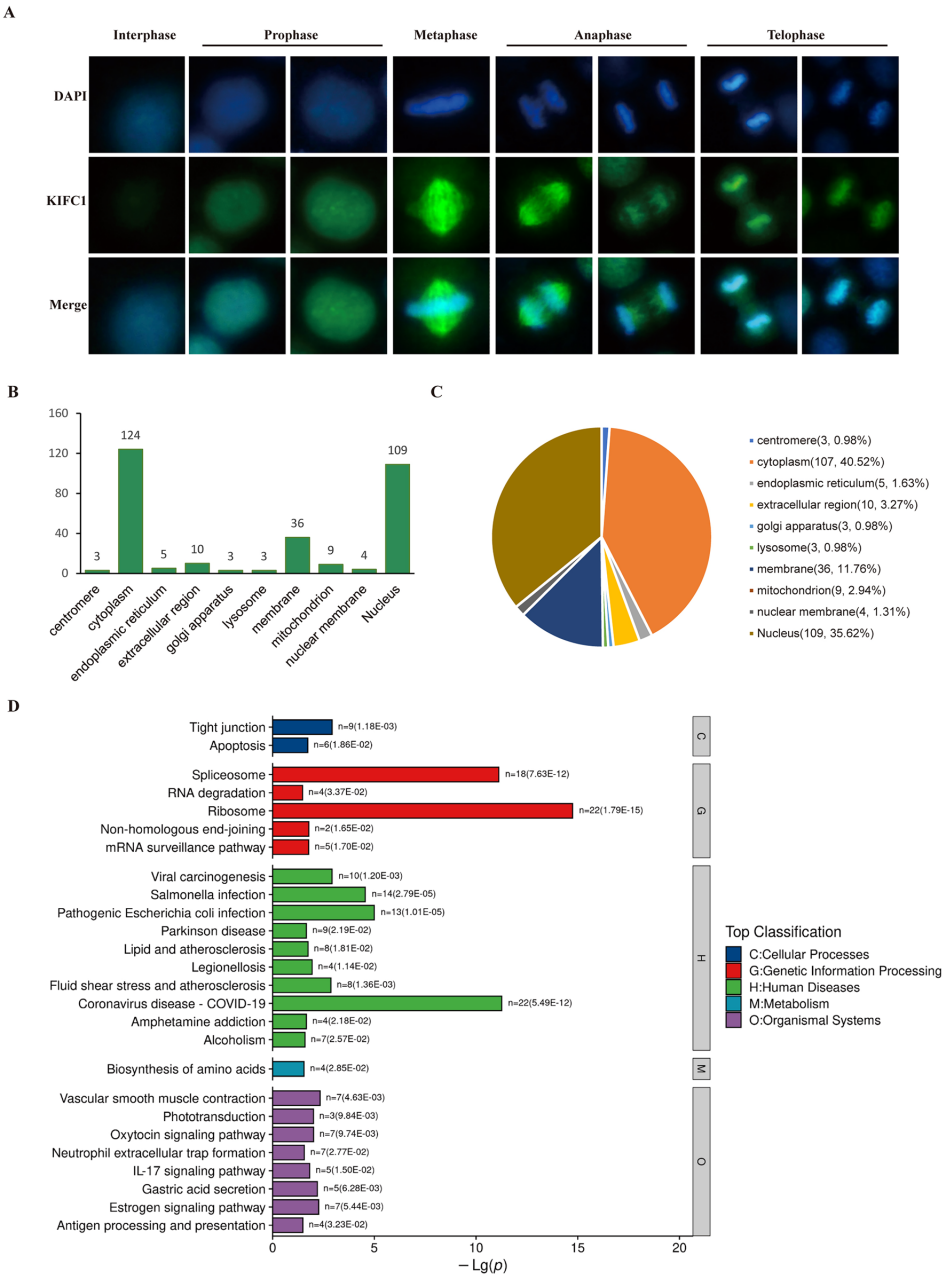
Analysis of proteins interact with KIFC1 (**A**) Immunofluorescence staining of KIFC1; (**B**) Subcellular localization analysis of KIFC1 binding proteins; (**C**) Statistics of subcellular localization; (**D**) GO analysis of KIFC1 binding proteins.

**Figure 9:**
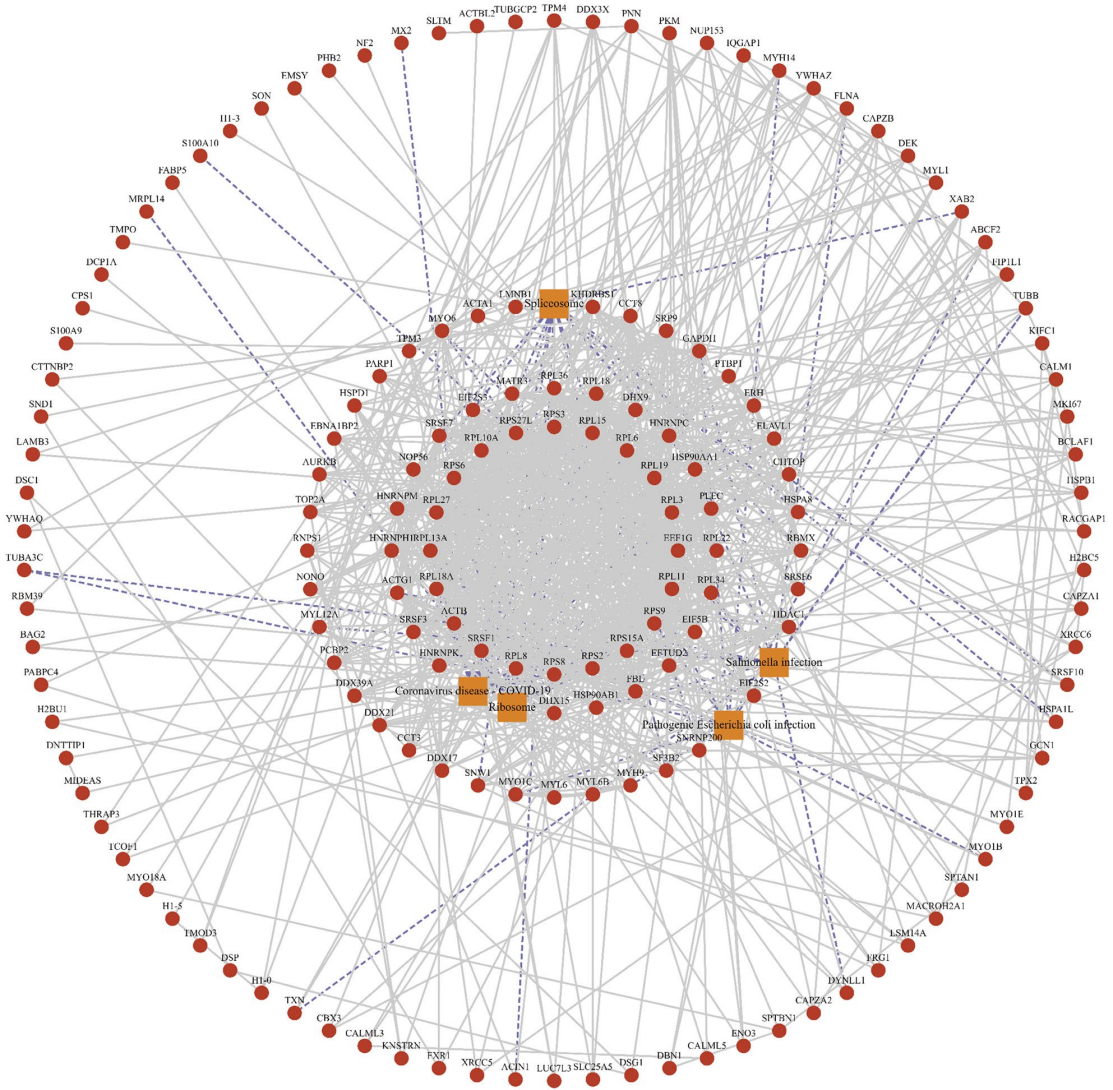
PPI analysis (concentric) of KIFC1 binding proteins.

In conclusion, during different cell cycle stages, KIFC1 mainly localized to the nucleus and spindle (Fig. 8A). Considering that KIFC1 is a molecular motor protein, we identified proteins with direct or indirect interaction with KIFC1 through co-immunoprecipitation and proteomics. The results showed that KIFC1 interacted proteins distributed very widely, with 40.52% of all proteins localized to the cytoplasm, 11.76% to the membrane, 35.62% to the nucleus, and 1.31% to the nuclear membrane (Fig. 8B,C). Major genetic information processes are spliceosome and ribosome (Fig. 8D). PPI analysis indicates interaction relationships and weights between candidate proteins (Fig. 9).

## Discussion

KIFC1 is expressed highly in a variety of tumors. Many studies suggest that KIFC1 could be used as a potential actionable biomarker of early-stage tumorigenesis and progression of high-risk lesions^14^. However, the protein expression of KIFC1 in some tumors was not elevated significantly from that in normal tissues. It was unsuitable for tumor markers, such as lung, colon, and cervical cancer^15^. The mRNA expression of KIFC1 was significantly higher in clear cell renal cell carcinoma than in healthy tissue; KIFC1 mRNA expression increased significantly with the increase of tumor grade. In terms of clinical stage, the mRNA expression of KIFC1 in clinical stage IV tumors was significantly higher than that in other stages, mainly due to the significantly elevated mRNA expression of KIFC1 in the metastases sample. When performing the prognostic analysis of tumors, the quartile grouping method achieved more significant differences than the method of bisection. The results indicated a significant prognostic difference between the top 25% of samples with mRNA expression of KIFC1 and the remaining samples. Further, we found that KIFC1 is an independent prognostic factor in renal clear cell carcinoma, implying that high expression of KIFC1 has an obvious prognostic value in patients with renal clear cell carcinoma.

KIFC1 has dual functions of material transport and microtubule organization. In oocytes, KIFC1 deletion leads to spindle instability and aneuploidy production^7,16,17^. The exact mechanism for maintaining spindle stability also is found in cisplatin resistance^4^ and neuronal migration^17^. However, KIFC1 also promotes tumor cell proliferation in other ways. For example, in liver cancer, KIFC1 enhances the transcriptional activity of HMGA1, thereby accelerating the expression of downstream genes^8^. Additionally, during the interphase, KIFC1 located in the nucleus. Overexpressed KIFC1 could significantly shorten the S phase's duration and promote the cell cycle's transition^5^. These are consistent with the results of our bioinformatics analysis, which showed a very significant positive correlation between the expression of KIFC1 expression and DNA replication, G2/M checkpoint, and tumor proliferation signaling pathways. However, our analysis revealed that KIFC1 expression was also strongly associated with tumor inflammation, which is associated with tumor metastasis, invasion, and even immunotherapy^18^.

In the progression of RCC, dramatic changes in cellular metabolic pathways have been observed, which are considered a key characteristic of RCC^19^. Our research has found a significant correlation between the overexpression of KIFC1 and many metabolic pathway changes in RCC, such as the downregulation of oxidative phosphorylation, lipid metabolism, and so forth, which are believed to play important roles in the development of RCC^20,21^. In addition, relevant research has demonstrated that elevated KIFC1 can downregulate reactive oxygen species in cells by enhancing glutathione metabolism, promoting tumor cell proliferation^22^, and facilitating the glycolytic pathway by regulating c-myc^6^. Mechanistically, we observed a significant positive correlation between KIFC1 and the core metabolic regulation signaling pathway, PI3K/AKT/mTOR^23^. Through co-IP, we discovered the translocation of KIFC1 to splicing body proteins (such as hnRNPM, which has been reported to regulate the PI3K/AKT/mTOR signaling pathway^24^. These results are exciting and warrant further in-depth investigations.

RCC is one of the tumors with the highest immune infiltration^25,26^, yet the underlying mechanisms remain unclear. Our research reveals a significant positive correlation between high KIFC1 expression and various metabolic pathways, which are believed to endow the tumor with stronger immune infiltration and a more complex immune microenvironment^27^. As seen in Fig. 6, a greater variety and quantity of immune cells, particularly CD8T, CD4T, and macrophages, were observed in tumors with high KIFC1 expression.

We analyzed immune cells within tumors and found that the amount of tumor-associated M2 macrophages^28^ was not significantly different in tumors with high KIFC1 expression. In contrast, the amount of M1 macrophages upregulated significantly (Fig. 6). it led us to focus our investigation on intratumoral T cells.

CD8 T cells can selectively detect and eradicate cancer cells^29^. T cells in late-progressing tumors become unresponsive, also known as dysfunctional, due to continuous exposure to tumor antigens^30^. Tumor-specific CD8 T cell hyporesponsiveness is a major mechanism of tumor evasion^31^. Intratumoral differentiation of T cells at progression to a more severe hyporesponsive state^31^. Early or late dysfunctional tumor-specific T cells could distinguish by surface protein expression^32^; PD1 and Lag3 are expressed in early-stage dysfunctional T cells^33^. Late-stage dysfunctional T cells express additional inhibitory receptors, such as CD38, CD39, CD101, and TIM3^34^. In tumors with high expression of KIFC1, the marker genes of immune dysfunction in the late stage of the tumor significantly increased. It suggested that there is a significant correlation between the high expression of KIFC1 and the functional loss of CD8 + T cells (Fig. 6A).

The tumor-infiltrating lymphocytes (TIL) population consists of tumor-reactive T cells and nontumor-reactive bystander T cells^35^. The proportion of tumor-reactive T cells varies widely among cancer types and patients. However, increased immune infiltration in tumors was thought to correlate with better outcomes and improved ICB therapy response^36^. High tumor immune infiltration does not necessarily correlate with increased tumor reactivity; only a tiny fraction of intratumoral CD8 T cells (approximately 10% or less) can recognize cancer cells^37^. Bystander T cells may express inhibitory receptors, including PD1, LAG3, CD39, TOX, and TIGIT, but they often lack features of chronic antigen stimulation^30^. We further analyzed the relationship between the expression of bystander T cell marker genes Lag3, TOX, TIGIT, and KIFC1 presence (Spearman coefficient > 0.7). These results suggest that the reason why tumor with high expression of KIFC1 is not sensitive to ICB treatment may be due to immune dysfunction (Fig. 7D).

PD1 expression is not specific for tumor-reactive T cells^38^. Accordingly, we also analyzed marker genes missing in bystander tumor cells, such as CD39 and CD103. Although they also increased significantly in the KIFC1 high expression group, the correlation between them and the expression of CD4 and CD8 genes was very poor. CD39 was another biomarker to distinguish tumor-reactive TILs from non-tumor-reactive bystander TILs^39,40^; In addition, the expression of CD103 is associated with increased tumor survival, and double-positive of CD103 and CD8 TILs indicated a better prognostic marker than total CD8 TILs^41^.

CD4T cells display a variety of functionalities, inhibiting tumor progression through either CD8-dependent or independent mechanisms^42^. Our study reveals a similar positive correlation between CD4T cells and KIFC1 expression (Fig. 7). Although the quantity of CD4T cells increases, these cells continue to exhibit a significant positive correlation with the expression of various immune checkpoint proteins. This implies that within the tumor, CD4T cells may also be in a state of exhaustion. However, the mechanism of CD4T cell exhaustion is not fully understood. Various human-based studies have confirmed the presence of exhausted CD4T cells within tumors, characterized by the expression of inhibitory receptors and dysfunctional attributes. Partial functioning can be restored with the application of checkpoint inhibitors^43,44^. Unlike terminally exhausted CD8 T cells, however, terminal exhaustion in CD4 T cells is represented by CD39 expression rather than TIM. CD39 positive cells have higher levels of PD-1, produce fewer cytokines, and are more likely to generate single-cell cytokines (mainly IFNγ) rather than co-producing multiple cytokines^42^. Evidence of transcription factor expression and epigenetic variations suggests the concurrent exhaustion of CD4T and CD8T cells within tumors. In comparison to CD8 exhaustion, tumors with exhausted CD4T cells display a stronger immune evasion phenomenon^45^.

In conclusion, we identified KIFC1 as an independent prognostic factor in renal clear cell carcinoma. Mechanistically, KIFC1 is significantly associated with cell proliferation, inflammation, and other signaling pathways. High expression of KIFC1 positively correlated with infiltration of tumor cells as well as dysfunction of T cells significantly. It was predictive of poor prognosis in clear cell renal cell carcinoma. By proteomics, we identified proteins that showed interaction with KIFC1. KIFC 1 may regulate the progression of RCC by regulating cell proliferation, immune infiltration, and ICB therapy, which is of great research value.

## Supplementary Information

Supplementary Information.

## Data Availability

Data used in this paper can be downloaded from the Firehose Legacy, https://portal.gdc.cancer.gov/projects/TCGA-KIRC.
