# Author Correction: Identification of KIFC1 as an independent prognostic marker in renal clear cell carcinoma correlates with tumor proliferation and immune infiltration

**DOI:** 10.1038/s41598-025-87056-x

**Published:** 2025-01-29

**Authors:** Bin Du, Jia Wang, Jinping Zheng, Jing Huo, Pu Wang

**Affiliations:** 1 Center of Healthy Aging, Changzhi Medical College, Changzhi, 047500 China; 2 Department of Biology, Changzhi Medical College, Changzhi, 047500 China

Correction to: *Scientific Reports* 10.1038/s41598-023-43732-4, published online 03 October 2023

The original version of this Article contained errors.

Due to an error during figure assembly, in Figure 8A two cells in the stage “telophase I” were selected. One exemplary cell has been replaced with a “telophase II” stage cell.

In addition, due to errors when assigning the protein localization from the LS/MS data in Figure 8B and 8C, some proteins were wrongly categorized. The data was reanalysed and the proteins were assigned their correct localization.

Consequently, in the Result section, under the subheading ‘Identifying proteins interact with KIFC1’,

“The results showed that KIFC1 interacted proteins distributed very widely, with 32.33% of all proteins localized to the cytoplasm, 23.69% to the membrane, 9.63% to the nucleus, and 2.01% to the nucleus (Fig. 8B,C).”

now reads:

“The results showed that KIFC1 interacted proteins distributed very widely, with 40.52% of all proteins localized to the cytoplasm, 11.76% to the membrane, 35.62% to the nucleus, and 1.31% to the nuclear membrane (Fig. 8B,C).”

The original Figure 8 and accompanying legend appear below.

**Fig. 8:**
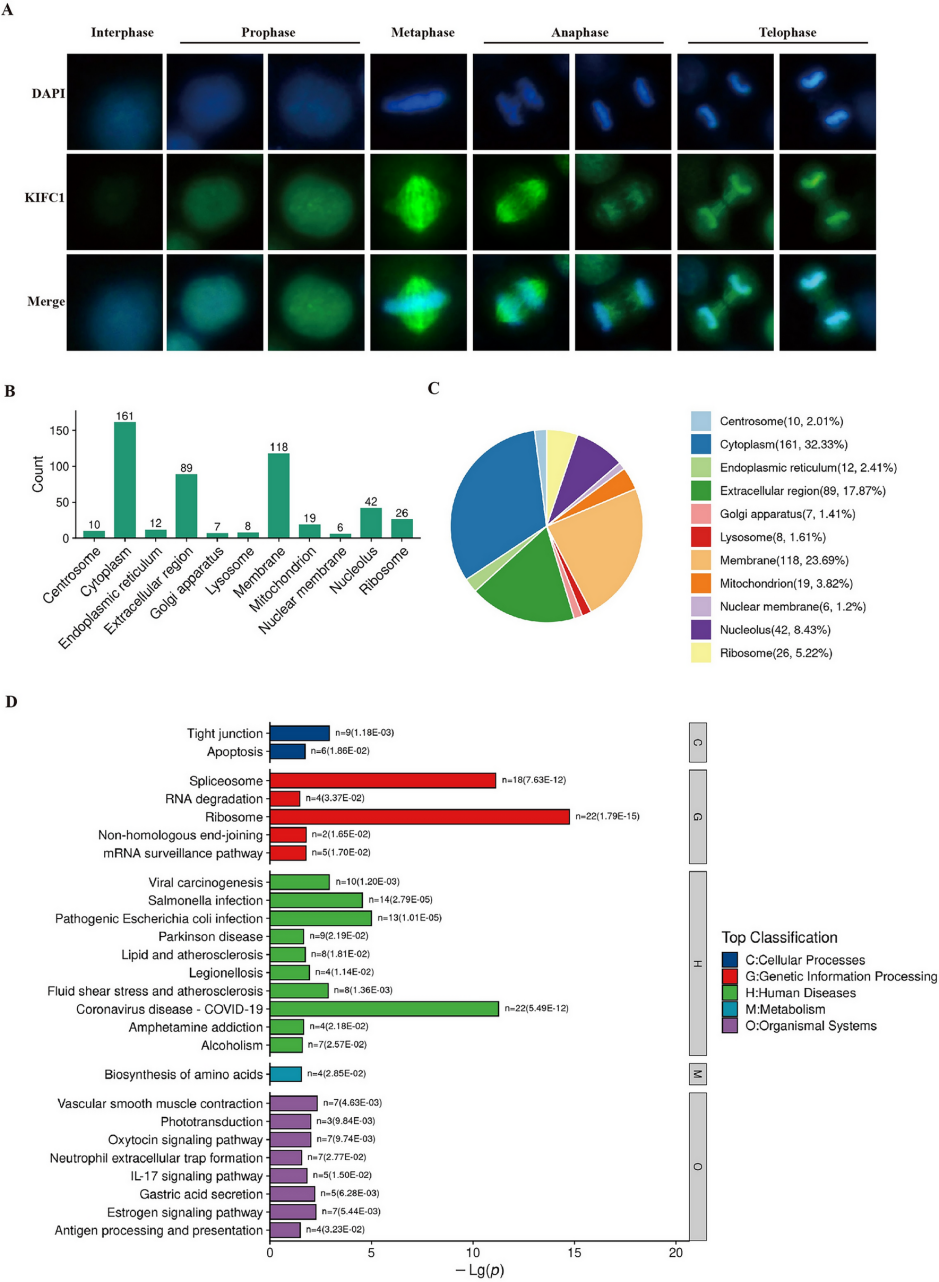
Analysis of proteins interact with KIFC1 (**A**) Immunofluorescence staining of KIFC1; (**B**) Subcellular localization analysis of KIFC1 binding proteins; (**C**) Statistics of subcellular localization; (**D**) GO analysis of KIFC1 binding proteins.

The original Article has been corrected.

