# Supplementary material for: Identification of KIFC1 as an independent prognostic marker in renal clear cell carcinoma correlates with tumor proliferation and immune infiltration
Source: Sci Rep. 2023 Oct 3;13:16572. doi: 10.1038/s41598-023-43732-4 (PMC10547834; doi:10.1038/s41598-023-43732-4)
Supplement: Supplementary file 1 — Supplementary Information. [file 41598_2023_43732_MOESM1_ESM.pdf]

Attachment: the original pictures of Figure 8A

Identification of KIFC1 as an independent prognostic marker in renal clear cell carcinoma correlates with tumor proliferation and immune infiltration

Bin Du[1, 2], Jing Huo[3], Jinping Zheng[1], Pu Wang[1, 3]*

1 Center of healthy aging, Changzhi Medical College, Changzhi 047500, China

2 Department of parasite, Changzhi Medical College, Changzhi 047500, China

3 Department of biology, Changzhi Medical College, Changzhi 047500, China

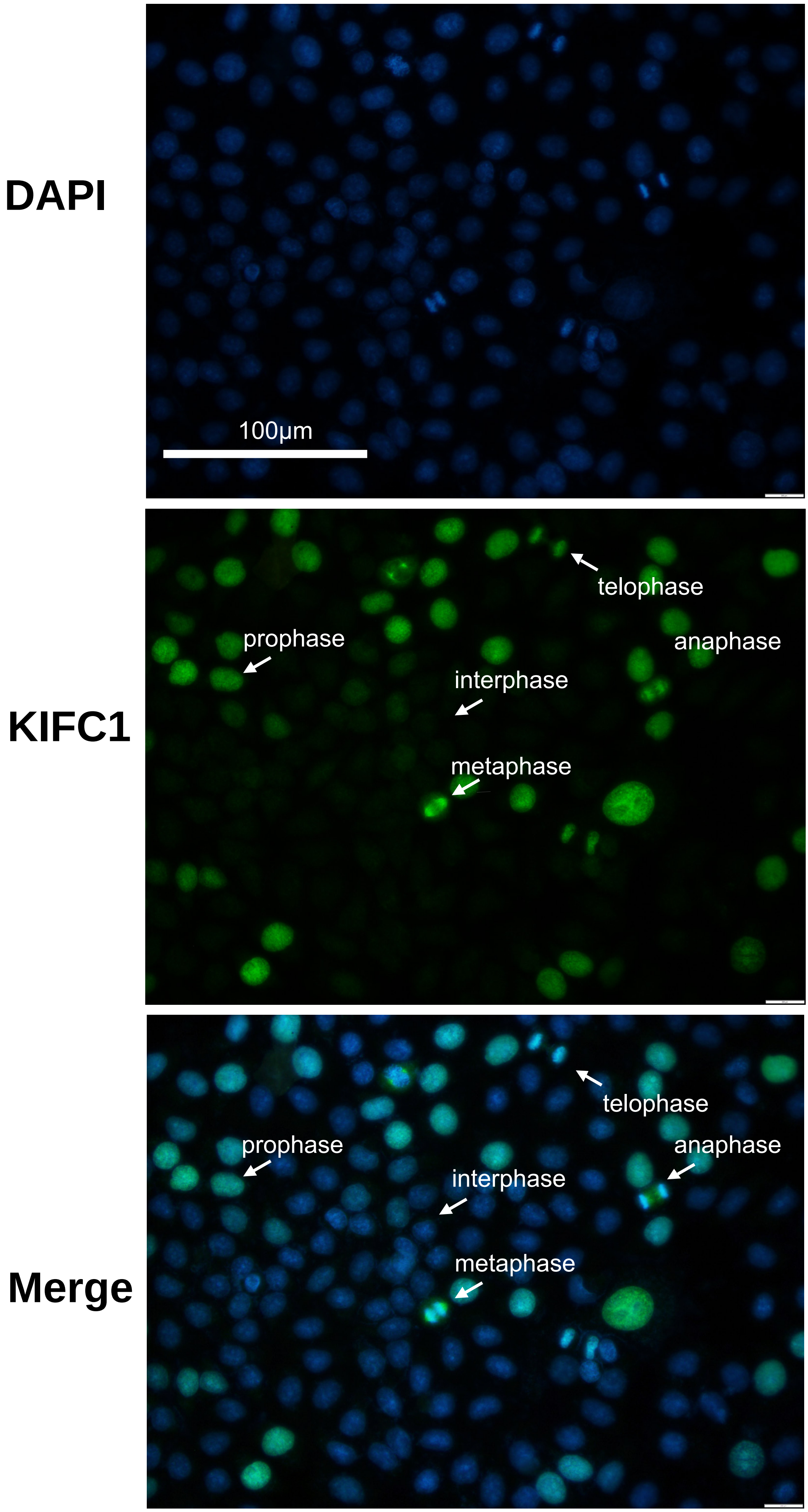
DAPI
100μm
KIFC1
telophase
prophase
anaphase
interphase
metaphase
Merge
telophase
prophase
anaphase
interphase
metaphase

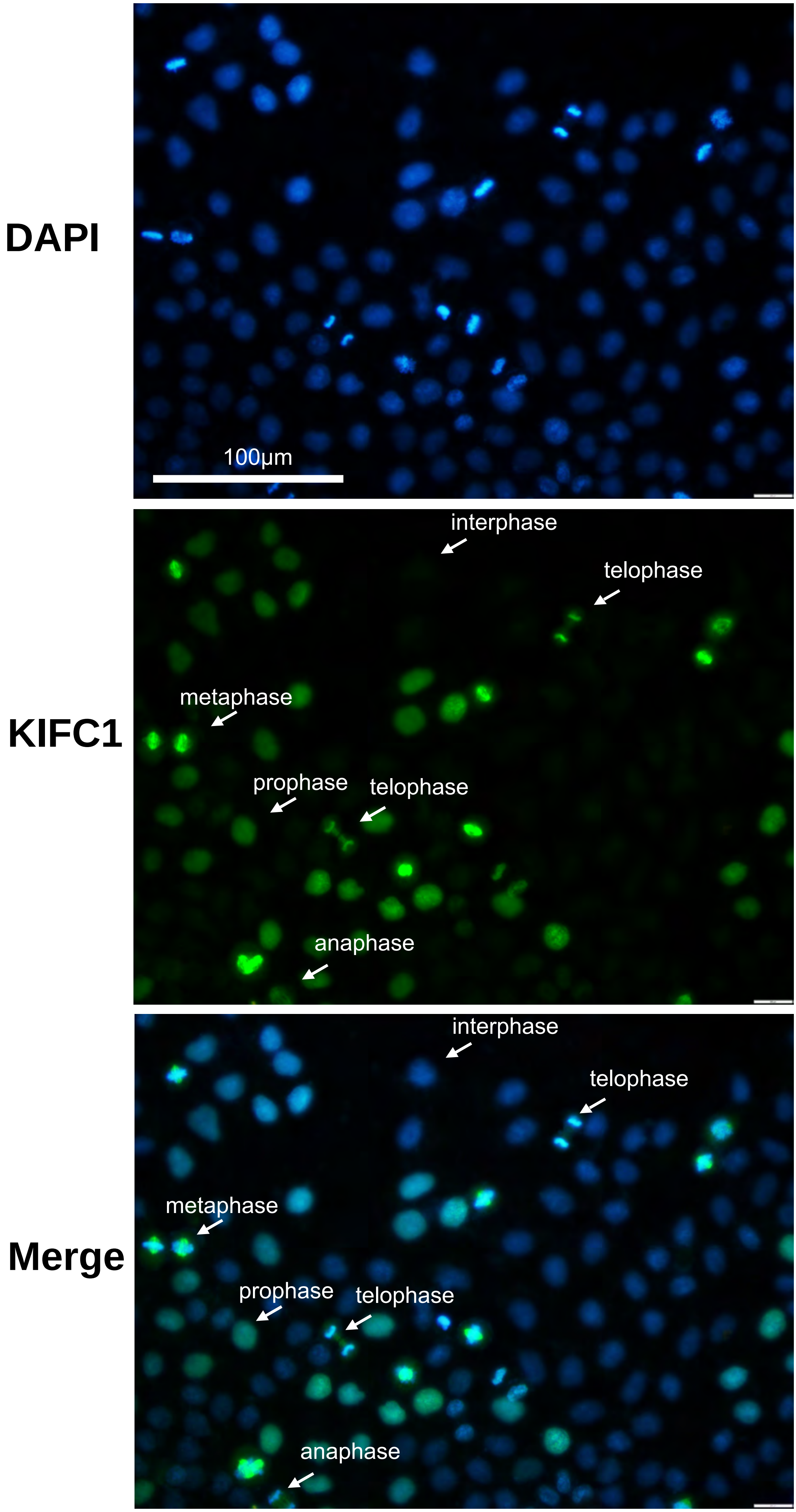
DAPI
100μm
KIFC1
interphase
telophase
metaphase
prophase
telophase
anaphase
Merge
interphase
telophase
metaphase
prophase
telophase
anaphase
